# The patterns of birthmarks suggest a novel population of melanocyte precursors arising around the time of gastrulation

**DOI:** 10.1111/pcmr.12645

**Published:** 2017-10-13

**Authors:** Veronica A. Kinsler, Lionel Larue

**Affiliations:** ^1^ Genetics and Genomic Medicine UCL Institute of Child Health London UK; ^2^ Paediatric Dermatology Great Ormond Street Hospital for Children NHS Foundation Trust London UK; ^3^ Institut Curie INSERM U1021 Normal and Pathological Development of Melanocytes PSL Research University Orsay France; ^4^ Univ Paris‐Sud Univ Paris‐Saclay CNRS UMR 3347 Orsay France; ^5^ Equipe Labellisée Ligue Contre le Cancer Orsay France

**Keywords:** birthmark, development, melanoblast, melanocytic, migration, mosaicism, non‐segmental, pattern, population, segmental, spray can, vitiligo

## Abstract

Systematic work in the mouse and chicken has mapped out two neural crest‐derived pathways of melanocyte precursor migration. With these in mind, this study reappraises the patterns of congenital pigmentary disorders in humans and identifies three recurrent patterns consistent across genetically different diseases. Only two of these are seen in diseases known to be melanocyte cell‐autonomous. The segmental pattern correlates well with the classical dorsolateral population from animal studies, demonstrating respect of the midline, cranio‐caudal axial mixing, unilateral migration and involvement of key epidermally derived structures. Importantly however, the melanocyte precursors responsible for the non‐segmental pattern, which demonstrates circular, bilateral migration centred on the midline, and not involving key epidermally derived structures, have not been identified previously. We propose that this population originates around the time of gastrulation, most likely within the mesoderm, and ultimately resides within the dermis. Whether it contributes to mature melanocytes in non‐disease states is not known; however, parallels with the patterns of acquired vitiligo would suggest that it does. The third pattern, hypo‐ or hyperpigmented fine and whorled Blaschko's lines, is proposed to be non‐cell‐autonomous.

## 1 INTRODUCTION

Extensive and systematic work in the mouse and chicken over the last 50 years has studied the pathways of melanocytic precursor migration (Bonaventure, Domingues, & Larue, 2013; Mort, Jackson, & Patton, 2015). Two major pathways of migration have been identified and characterized, both originating from the neural crest. The first is the classical dorsolateral pathway in both mouse and chick, in which melanoblasts delaminate from the neural crest and migrate between the developing somites and overlying dermis (Erickson & Goins, 1995). The second is the more recently described dorsoventral pathway in the chick, in which melanoblasts develop from Schwann cell precursors in association with peripheral nerves (Adameyko et al., 2009). A key feature of both these pathways is a dorsal midline separation of melanocytic precursors into right or left halves of the embryo. In the dorsolateral pathway, there is fate specification before migration, whereas in the dorsoventral pathway, the melanocytes develop after the initial migration of a multipotent Schwann cell precursor. Recent ex vivo imaging and mathematical modelling of the murine dorsolateral melanoblast population has concluded that melanoblast migration is by processes of random diffusion and proliferation coupled with embryonic growth, rather than by directed dorsoventral migration (Mort et al., 2016).

In humans, although there is no reason to believe that melanocytic development should be any different, it is worth noting that dynamic studies of migration in early embryogenesis have not been possible for ethical reasons, and therefore, the small amount of data available has been largely gleaned from static immunohistochemical studies from the age of 6 weeks onwards (equivalent to E11 in mice). From these data, human melanocyte precursors appear to enter the epidermis at week 6 and are clearly distinguishable by HMB‐45 positivity (which detects gp100/PMEL) by seven weeks of gestation [E12.5 mouse] (Holbrook, Underwood, Vogel, Gown, & Kimball, 1989). Later, HMB‐45‐positive cells are abundant in the epidermis and are still present in the developing dermis throughout 3–7 months of gestation [E13.5–E18.5 mouse] (Luciani et al., 2011; Suder & Bruzewicz, 2004). Mature perifollicular and intrafollicular epidermal pigmented melanocyte populations are maintained in post‐natal life, located at the basal layer of the epidermis, and mature melanocytes have also been found in sebaceous glands (Jang et al., 2014). There is also evidence for the common presence of dermal melanocytes in children of at least some ethnicities up to the age of 10 years, in the form of the common non‐pathogenic pigmentation Mongolian blue spots (also known as dermal melanocytosis), and indeed some evidence of persistence throughout life (Kikuchi & Inoue, 1980).

Distinct melanocyte stem cell populations have been reported and characterized in adult mice and humans, with specific hair follicle‐associated and skin‐derived dermal stem cells both exhibiting capability for differentiation into melanocytes in vitro (Amoh, Li, Katsuoka, Penman, & Hoffman, 2005; Fernandes et al., 2004; Glover et al., 2015; Nishikawa‐Torikai, Osawa, & Nishikawa, 2011; Nishimura, 2011; Osawa et al., 2005; Schneider, Dieckmann, Rabe, Simon, & Savkovic, 2014; Yu, Kumar, Kossenkov, Showe, & Xu, 2010; Yu et al., 2006). There are, in addition, dermal stem cells in glabrous skin, which is devoid of hair follicles (Li et al., 2010), and in sweat glands (Grichnik et al., 1996; Okamoto et al., 2014), both capable of differentiation into melanocytes in culture. The renewal of mature melanocytes is presumed to occur from these stem cell populations, with recent evidence demonstrating that the non‐follicle‐associated stem cells do indeed contribute to the interfollicular melanocyte population, at least in the mouse (Glover et al., 2015). What is not known, however, is whether these different melanocyte stem cell populations have different embryological origins, and whether they maintain different mature melanocyte populations. Importantly, it is conceivable that an improved understanding of these factors might lead to an improved understanding of acquired melanocytic diseases such as vitiligo or melanoma.

In the absence of an approved ethical method to study human melanocytic development systematically under laboratory conditions, it is possible to use a more traditional tool in the clinical academic's armoury—the study of the patterns of rare congenital diseases of pigmentation (relevant examples of which are outlined in Supplementary Material). Patterns of these diseases have been described phenotypically for many years (Blaschko, 1901; Happle, 1993; Torrelo, Baselga, Nagore, Zambrano, & Happle, 2005); however, many descriptions were undertaken before knowledge of the underlying genetics and of the most recent findings in embryology. In particular, until recently there were limited data on which conditions were definitely due to post‐zygotic mutations leading to mosaicism and in which clonality of pigmentary patterns has been proven at the level of the founder mutation. This study therefore set about reappraising patterns and their implications in the light of the latest molecular and embryological data.

## 2 METHODS

Given the number and complexity of patterns of human congenital pigmentary disorders, some premises and hypotheses were established for pattern interpretation.

### 2.1 Premises and hypotheses on which pattern interpretation is based in this study

Firstly, a small single birthmark can occur in any place due to a late post‐zygotic mutation of any cell type in the skin. These are therefore not informative for developmental pattern interpretation and are not classifiable on that basis. Interpretation was focused therefore on multiple (two or more of any size or site, where the same causative mutation has been shown for that diagnosis to be present in both birthmarks) or extensive birthmarks (estimated diameter of more than 10 cm at birth).

Secondly, it is proposed that congenital pigmentary patterns may represent persistent embryological or foetal developmental patterns, which do not necessarily correspond to post‐natal patterns of cellular organization. This is supported by evidence from systematic studies of other birthmarks, notably infantile haemangiomas (Haggstrom, Lammer, Schneider, Marcucio, & Frieden, 2006; Weitz et al., 2014) and capillary malformations (Waelchli et al., 2014), the patterns of which correspond to the fields of the embryonic facial placodes, rather than to adult vascular supply.

Thirdly, post‐zygotic or mosaic clonal disorders can be particularly powerful at revealing the patterns of normal development. This is because the pattern visualized is of necessity limited to the distribution covered by the offspring of a single precursor cell in that disease state. The patterns in these disorders therefore permit mapping of the maximal individual melanocyte precursor fields at a time when that mutation is compatible with survival of the embryo. Smaller areas of abnormal pigmentation should fit within any one of these fields, representing a later (although usually identical) mutation in embryogenesis.

Fourthly, where it is possible to identify recurrent congenital patterns of pigmentation in mosaic clonal diseases of known and distinct genetic aetiology, we can reasonably assume that the causal mutations do not alter the final melanocyte precursor “fields”—in other words, the area of the skin covered by melanocytes from a single melanocyte precursor—even if the numbers or morphology of the cells is altered by the disease state. Such diseases therefore allow us to visualize the patterns and extents of normal melanocytic precursor fields, effectively frozen in the embryonic distribution not usually visible in the refined and remodelled phenotype of cutaneous pigmentation seen by the time of birth. Conversely, very rare patterns of pigmentation, which are not yet known to be shared by more than one genetically different disease, may be secondary to a genetic abnormality which interferes with melanocyte precursor migration. These include the sash‐like pattern of Ruggieri–Happle syndrome (Ruggieri, 2000), the disordered swirling linearity of Pallister–Killian syndrome and phylloid patterns (Happle, 1993). As a result, these were not considered alongside the recurrent patterns in this work.

Fifthly, we did not assume that all congenital patterns of pigmentation on the skin are melanocyte cell‐autonomous. When known melanocytic disorders are not seen in certain patterns, these patterns are more likely to be due to primary abnormalities of other cell types (e.g., keratinocytes) and are visible by the effect they have on the pigmentary system.

Lastly, where congenital pigmentary disorders consistently spare or involve structures of known embryological lineage and developmental timing, these data can be used to formulate hypotheses about the origin of melanocytes in those structures, for example, structures which arise in situ from the surface ectoderm or epidermis, such as the palpebral fissures of the eyelids and the eyelashes (from the surface ectoderm in the 10th–11th weeks of gestation; Andersen, Ehlers, & Matthiessen, 1965), the areola of the nipple (from the epidermis in approximately the fifth month of gestation; Hughes, 1950), and the vermilion border of the lips (from the surface ectoderm overlying the component facial placodes after the eighth week; Coslet & Cohen, 1967; Warbrick, 1960). As melanocytes from the neural crest are already within the epidermis from 8 weeks, clonal pigmentary patterns which involve these epidermal structures could be considered to have arisen from these neural crest melanocytes, whereas those which spare these structures could be considered to have arisen independently of the neural crest melanocytes, or at least independently from those which reached the epidermis.

### 2.2 Resources used

With these premises in mind, a systematic review was undertaken of 6,405 photographs of 1,229 patients seen in the Paediatric Dermatology department at Great Ormond Street Hospital for Children between 2002 and 2015 with a diagnosis of any congenital pigmentary disorder affecting the skin. In addition, all hospital photographs of vitiligo were reviewed, and a comprehensive review undertaken of published photographs of pigmentary disorders in textbooks, journals and online. The most extensive recurrent fields of each pattern in mosaic disorders were identified. Attention was paid to patterns that might be expected but were never seen, such as fine and whorled Blaschko's lines in known melanocyte cell‐autonomous disorders.

The criteria for deciding whether a pattern of pigmentation was melanocyte cell‐autonomous were whether it has been seen when the genetic abnormality is proven to affect melanocytes, or where the abnormal cells are highly likely to be melanocytic cells (such as naevus cells). Included in this definition were the post‐zygotic mosaic conditions: multiple congenital melanocytic naevi (CMN, usually caused by *NRAS* mutations; Kinsler et al., 2016), phakomatosis pigmentovascularis and extensive dermal melanocytosis (PPV and EDM, respectively, usually caused by *GNA11/GNAQ* mutations; Thomas et al., 2016), phakomatosis pigmentokeratotica (PPK, usually caused by *HRAS* mutations; Groesser et al., 2013), congenital naevus spilus (caused by HRAS or NRAS) (Kinsler et al., 2014; Sarin et al., 2013), McCune–Albright syndrome (MAS, caused by *GNAS* mutations; Weinstein et al., 1991) and mosaic Neurofibromatosis type 1 (NF1, caused by *NF1* mutations (Ainsworth, Chakraborty, & Weksberg, 1997); see Supplementary Material for further details of these conditions). Of note, the pigmentary patterns in these mosaic disorders have been found and published to be clonal at the level of the causative mutation in multiple patients with the same diagnosis, even where there is geographical separation of pigmentary lesions on different body parts. In this large group of patients studied here, however, not every patient has been genotyped, although many have. In addition to these mosaic disorders, the patterns of the autosomal dominant condition piebaldism (germline *KIT* mutations; Fleischman, Saltman, Stastny, & Zneimer, 1991; Giebel & Spritz, 1991, where the phenotype is known to be cell‐autonomous; Mayer & Green, 1968), was studied, after definition of the fields using the mosaic disorders.

## 3 RESULTS

Three major recurrent patterns of congenital pigmentary disorders were identified. Where it was not possible to obtain consent for publication of specific features, references have been given which demonstrate these features in photographs. We propose that one of these patterns is melanocyte non‐autonomous, corresponding to fine and whorled Blaschko's lines (Blaschko, 1901; Happle, Fuhrmann‐Rieger, & Fuhrmann, 1984; Sorlin et al., 2017), and two are melanocyte cell‐autonomous. These two are here termed segmental and non‐segmental, in an attempt to link them to existing terminology, although their characteristics are redefined here. The segmental and non‐segmental patterns are proposed to represent distinct melanocyte precursor populations, on the basis that they are not seen together in clonal mosaic diseases in one individual, they have different defining characteristics (Table 1), and the fields of each pattern come together in a jigsaw‐puzzle‐like manner to cover more or less the entire integument in two different ways. The segmental pattern corresponds well to the classical neural crest dorsolateral population, whereas the non‐segmental population is currently unstudied in a laboratory setting.

**Table 1:** Comparison of the key phenotypic features of the two melanocyte cell‐autonomous patterns of congenital pigmentary disorders and an estimation of melanocyte precursor numbers at a time in development when the pigmentary abnormalities can exist without necessary abnormalities of other organ systems

	Segmental—”quadrilateral shapes”	Non‐segmental—”round shapes”
Pattern edges	Straight edges and linear patterns, which translates into parallel transverse borders across trunk and face, and linear edges along length of limbs	Convex‐outwards edges and therefore circular or ovoid patterns, which translates into rounded edges on trunk and head and transverse edges circumferentially around limbs
Pattern types	Band (bilateral), flag (unilateral), checkerboard (bilateral) and broad Blaschko‐linear (bilateral or unilateral) patterns	Cape, bathing trunk, glove‐and‐stocking, knee, elbow
Trunk/head/limb involvement	Continuity of truncal migration from midline linearly down the limbs.	Continuity of truncal migration in circular patterns includes the proximal limbs.
Cranio‐caudal axial mixing before embryonic migration	Yes	No
“Spray can” patterning	Little or no spray can patterning	Prominent spray can patterning—smaller pigmentary lesions in association with a larger one, or multiple small
Involvement of specific structures	Involves epidermal structures such as nipples, lips, palpebral fissures	Spares epidermal structures such as nipples, lips, palpebral fissures
Subdivisions	None	Dorsal and ventral divisions
Approximate number of proposed single melanocyte precursor fields	Total estimate 8–14 melanocyte precursor fields (Figure 1g,h, respectively, as this is a spectrum—numbers below are for 1h) *Trunk/Limb* 10 dorsal truncal/limb (5 each side of the dorsal midline which are contiguous with the ventral surface) *Head* Four for face and neck (two each side of midline, contiguous dorsal and ventral; Figure 1g)	Total estimate 12–13 melanocyte precursor fields (Figure 5) *Trunk* 2 dorsal truncal, contiguous dorsal and truncal (1 upper (Figures 2a,b,e and 5a) and 1 lower (Figures 2c,d,f and 5b; NB. in some mosaic clonal disorders, these two can be seen as a single field (Figure 2k,l), and must therefore arise from a single cell before cranio‐caudal division along the dorsal axis—this could therefore be counted as 1, and accounts for our estimate of either 12 or 13) Ventral truncal (1) (Figures 4a,d and 5j) *Limbs* 4 distal limb, contiguous dorsal and ventral (2 upper limb Figures 2g‐i and 5c; 2 lower limb Figure 2j‐n and 5d) 2 mid‐limb, contiguous dorsal and ventral (1 each lower limb; Figures 4b,c,e and 5k) *Head* 1 dorsal cranial midline scalp (Figures 1a,e,i and 5e) 3 ventral cranial (facial/anterior scalp, 1 frontonasal (Figures 1b,f,j and 5f), 1 mid‐face (Figures 1c,g,k and 5g) 1 chin (Figures 1d,h,l and 5h)
Overlapping melanoblast fields	No, none or very little overlap between fields from single melanocyte precursors, and no areas of paucity of coverage other than palms, soles and digit tips	Yes, substantial overlap between fields from single melanocyte precursors, and conversely also areas of paucity of coverage around mouth, eyes, chin, anterior/ventral trunk, knees, digit tips

### 3.1 Two recurrent patterns of congenital melanocyte cell‐autonomous pigmentary disorders

The key phenotypic features of the two patterns as defined here are given in Table 1. Where it was not possible to obtain consent for photographs of specific fields, references have been given to photographs in the literature.

#### 3.1.1 The segmental pattern—”quadrilateral shapes”

**Figure 1:**
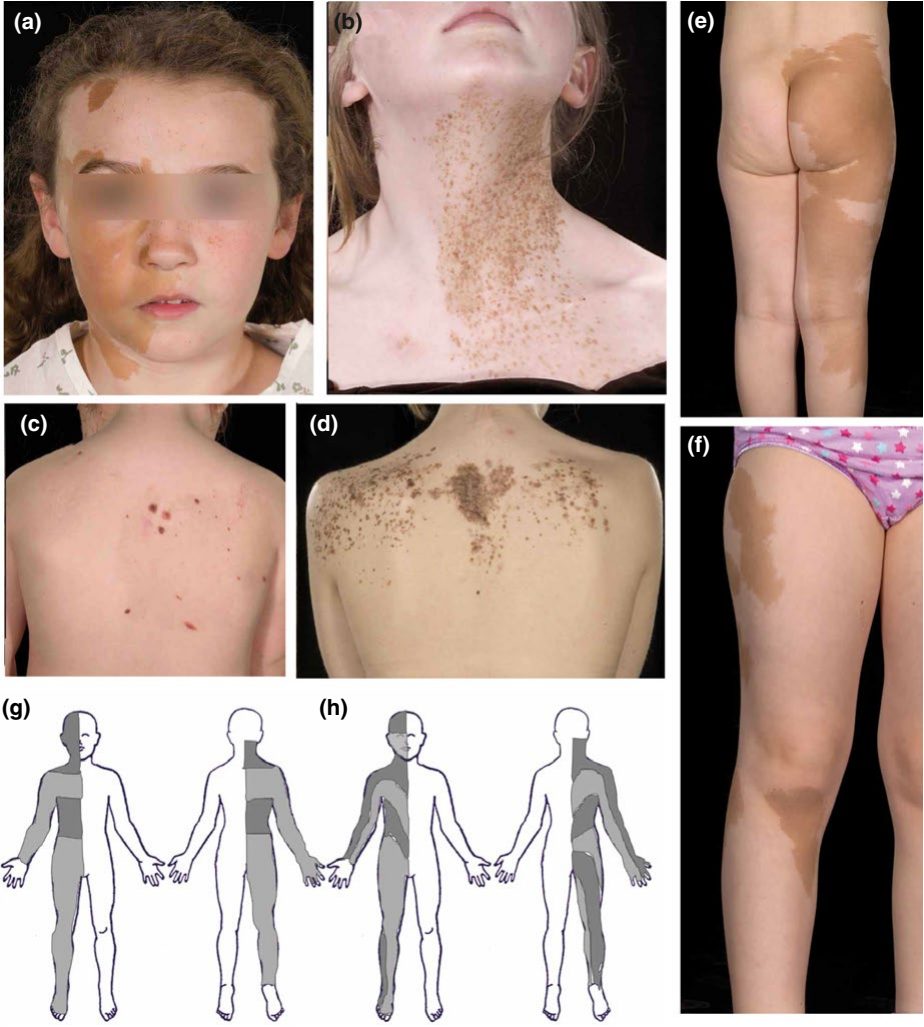
Segmental pattern of melanocyte cell‐autonomous congenital pigmentary disorders. (a) Café‐au‐lait macular pigmentation with midline demarcation corresponding to schematic 1h. (b) Macular naevus spilus on the neck and upper chest with midline demarcation representing less than a maximal single precursor field. (c) Papular naevus spilus on the back as part of phakomatosis pigmentokeratotica (PPK) in a checkerboard distribution, also involving the contralateral face with midline demarcation (not shown). (d) Papular naevus spilus as part of PPK in a band‐like distribution on the back, demonstrating the occasional bilateral occurrence of this pattern. (e,f) Café‐au‐lait macular pigmentation in a “broad Blaschko‐linear” pattern on the lower limb corresponding to 1h. (g,h) Schematic views of the approximate anterior/ventral and posterior/dorsal segmental pattern in its embryonically earlier large‐segment form (g) and later “broad Blaschko‐linear” form (h). The alternating pale and dark grey stripes demonstrate adjacent single melanocyte precursor fields and are contiguous dorsally and ventrally. NB. The patients shown in (a) and (e,f) do not have a clear diagnostic grouping. However, these examples are included here as consent was obtainable for publication, and this pattern is seen in the melanocytic diseases listed which produce large café‐au‐lait macules. Written consent was obtained for publication of all patient photographs. For a full description of the patterns, see the main text

This pattern is seen very commonly in naevus spilus (maculosus and papulosus), PPK and MAS, in congenital café‐au‐lait macules of NF1, regularly in EDM, and rarely in CMN. The pigmentation in this pattern covers nearly the whole integument, including that of epidermal structures such as the vermilion border of the lips, the eyelids including the palpebral fissures, the areolae of the nipples and excluding only the palms, soles, nail beds and fingertips. The characteristics of this pattern can be summarized as defined by straight edges, forming rough quadrilateral shapes. In the diseases studied, this pattern is often seen as crude divisions of the embryo into large cranio‐caudal segments of pigmentation (Figure 1g); however, it can be seen with smaller segmental divisions along the dorsal midline (Figure 1h), perhaps related to the increasing segmentation of the embryo. This is therefore a spectrum of segmental patterns, from what is known as flag‐shaped (Figure 1a–c, g) and checkerboard (Happle, 2014; Tadini et al., 1998; Figure 1g), to broad linear bands sometimes termed “broad Blaschko‐linear patterning” (Figure 1e,f,h; Happle, 2014). Notably, the segmental pattern spectrum does not seem to have areas of overlap between the fields derived from single melanocyte precursors, on the basis of mapping these fields onto an adult human body map. With extensive birthmarks, these segmental patterns include both the dorsal and ventral surfaces, whereas smaller birthmarks within these fields can affect only one or other. The segmental pattern for the scalp skin is not yet clear, as scalp hair obscures many of the photographs studied and further work will be needed to determine this.

The crude larger‐segmental pattern of pigmentation on the trunk is delimited by sharp transverse edges (Figure 1c,d,g). These are usually also delimited by the posterior/dorsal and/or the anterior/ventral midline, leading to a quadrilateral flag‐shaped pattern of unilateral blocks of pigmentary change, or a checkerboard pattern where multiple segments are affected at different cranio‐caudal levels (Biesecker & Spinner, 2013; Happle, 1993; Tadini et al., 1998). In some cases, the pattern can be bilateral, with transverse blocks either side of the midline at the same cranio‐caudal level, leading to a band‐like pattern at one or multiple cranio‐caudal levels (Figure 1d; Happle, 2014). In these large‐segment patterns, there appears to be four cranio‐caudal segments on either side of the midline, constituting eight segments in all (Figure 1g, Table 1). This segmental type of pattern is also seen in some of the melanocytic diseases (particularly MAS, and mosaic NF1) with a modest increase in numbers of segments to approximately 14 (Figure 1h, Table 1) and begins to take on a slightly more complex outline sometimes termed broad Blaschko‐linear patterning (Happle, 2014) (Figure 1e,f,h). Notably, this is distinct from fine and whorled Blaschko's lines (Blaschko, 1901; Happle et al., 1984), not having the complexity of the lines and whorls, but are rather a series of broad linear bands which show a midline dip.

#### 3.1.2 The non‐segmental pattern—”round shapes”

This pattern is seen commonly in CMN, EDM and PPV, and rarely in large congenital café‐au‐lait macules in NF1. The characteristics of this pattern can be summarized as defined by round edges, forming rough round or ovoid shapes. To demonstrate this pattern, we will focus on a series of skin areas which correspond to single melanocyte precursor fields, with the maximal extent of each field as identified using cases of CMN as a model. In total in this new population, we estimate there are approximately 12–13 founder melanocyte precursors (Table 1, Figure 5). There is however considerable overlap between the fields, based on mapping the fields onto an adult human body map (Figure 5). There are also areas of paucity with this population, particularly around the eyes, the mouth, to some degree on palms and soles, and at digit tips/nail beds (Figure 5l).

##### Dorsal non‐segmental pattern—trunk and head

**Figure 2:**
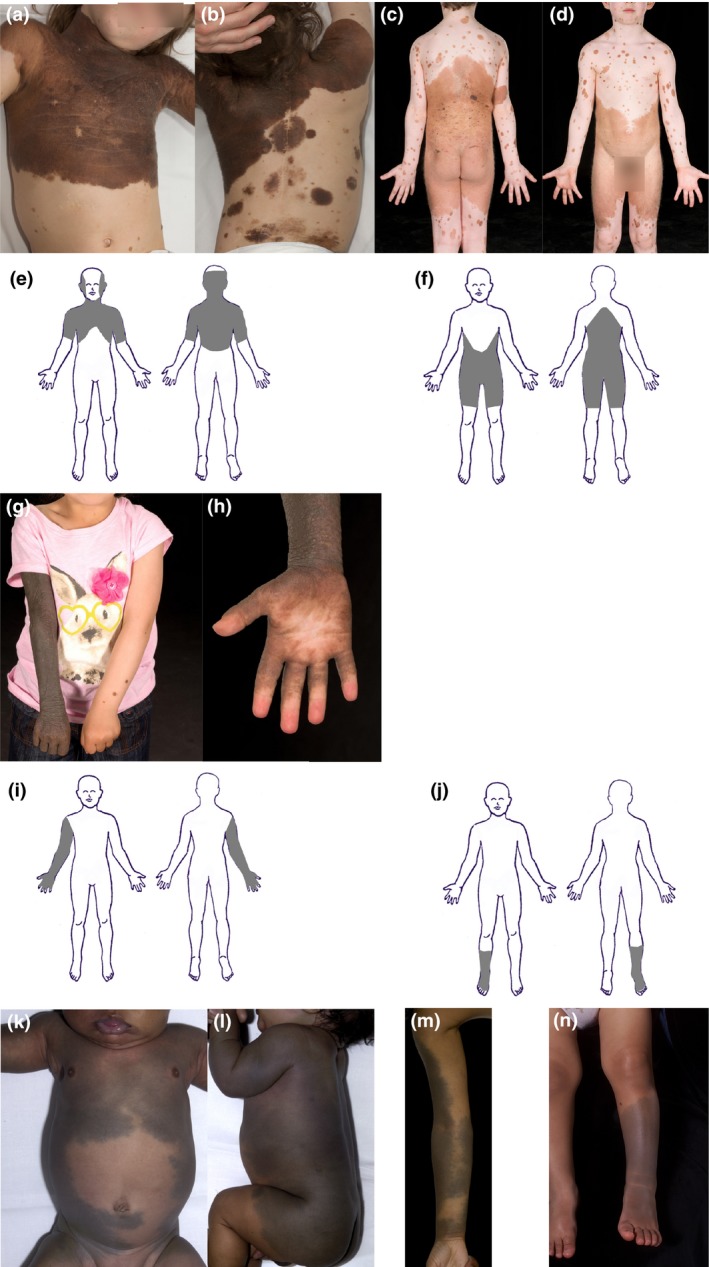
Dorsal non‐segmental pattern of mosaic and clonal pigmentary disorders at the level of the trunk and limbs. (a,b) Multiple congenital melanocytic naevi (CMN) in a “cape” distribution with a bilaterally roughly symmetrical distribution with convex‐outwards edges, corresponding to the schematic representation of the single melanoblast field in (e). (c,d) Multiple CMN in a “bathing trunk” distribution, corresponding to the schematic representation in (f). (g,h) Multiple CMN in a distal limb/”fingerless glove” distribution with involvement of the palm and sparing of digit tips and nails, demonstrating transverse cut‐offs circumferentially across the limb, and corresponding to the schematic representation in (i). (k) Schematic of the distal limb/”toeless stocking” field, demonstrating the same principles as in the upper limb (clinical picture not available for CMN). (k–n) Similar patterns seen in phakomatosis pigmentovascularis (PPV) and extensive or atypical dermal melanocytosis (EDM). (k,l) Occasional clonal mosaicism affecting both upper and lower truncal fields (cape and bathing trunk) suggesting that these two dorsal truncal fields were originally from a single cell. (m) Apparent dorsal to ventral development of the distal limb fields, with demonstration of convex‐outwards edges also on the limbs. (n) Sparing of digit tips in distal limb field in EDM. Written consent was obtained for publication of all patient photographs. For a full description of the patterns, see the main text

There are two dorsal truncal fields, upper and lower, which correspond to what has classically been described in clinical texts as “cape” (Figures 2a,b,e, 5a and 8e) and “bathing trunk” patterns (Figures 2c,d,f, 5b and 8f) on the posterior/dorsal skin surface. In EDM and PPV, these can be seen as a single field (Figure 2k,l). In very few published cases of CMN, these have been seen as a single field, and of note, these children also had epidermal abnormalities (Raina & Chaudhuri, 2010; Ramesh et al., 2017). The two dorsal truncal fields are bilateral and roughly symmetrical around the posterior/dorsal midline and include the proximal portions of the respective limbs. The cut‐offs of the dorsal truncal fields on the limbs are transverse and circumferential (Figure 2a–f,l), The cut‐offs are usually above the elbow and above the knee, but in rare cases, the cut‐off can be as far down as the wrist (Figure 8e) or to the mid‐calf.

The upper field extends from the scalp around the vertex, down the lateral edges of the face but sparing the central forehead and face, circumferentially onto the neck including under the chin, down onto the upper limbs circumferentially usually above the elbows, circumferentially onto the trunk as far down as the region of the lower lumbar vertebrae posteriorly/dorsally and below the nipples but above the umbilicus anteriorly/ventrally (Figures 2a,b,e, 5a, 6d, 7c,d and 8e). The circumferential anterior/ventral joining of this field is however very variable between individuals in all diseases studied, even with the maximal extent of the field affected on the posterior/dorsal axis, and the anterior/ventral surface of the trunk is often poorly supplied by this field. Posteriorly/dorsally, the lower border is usually convex downwards, with a slight upwards slope from posterior/dorsal to anterior/ventral, resulting in a transverse or concave‐upwards lower border on the anterior/ventral trunk (Figures 2a,b, 5a, 6d, 7c, 8e).

The lower truncal field is circumferential on the trunk, extending posteriorly/dorsally from the region of the upper thoracic vertebrae across the buttocks and perineum, and down onto the lower limbs circumferentially usually above the knees (Figures 2c,d,f, 5b and 8f). The upper border posteriorly/dorsally is convex upwards (Figures 2c, 5b, 6c,g and 8f), extending around onto the abdomen with a downward slope from posterior/dorsal to anterior/ventral on each flank, resulting in a concave‐downwards upper border anteriorly/ventrally, usually but not always dipping below the nipples (Figures 2d, 5b and 7b). Again, the anterior/ventral extension of this field is variable between individuals. The umbilicus can appear to be spared in this pattern (Figures 2d and 7a), but it is clearly involved if the anterior/ventral level extends high enough (Figure 7b).

**Figure 3:**
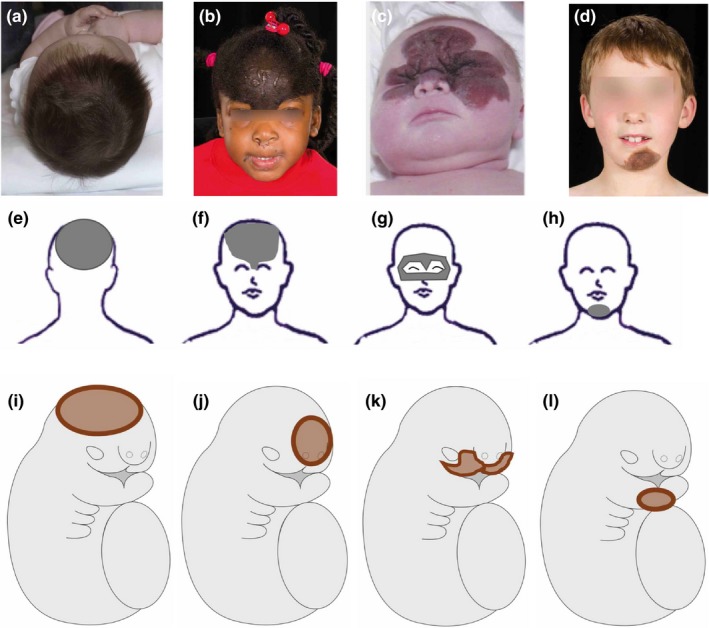
Non‐segmental patterns of pigmentary disorders at the level of the head. (a–d) Congenital melanocytic naevi (CMN) in distributions corresponding to the four bilaterally symmetrical midline cranial fields: (a) posterior/dorsal midline scalp, (d) ventral frontonasal, (g) ventral mid‐face and (j) ventral mandibular regions. (e–h.) Schematic representation of the four fields. (i–l) Schematic human embryo at the time of facial placode formation during week 4. Proposed fields (brown) of the four melanocyte precursors involved in the cranial non‐segmental pattern. These precursors may divide and diffuse in a radial way leading to a circular field of pigmentation. Written consent was obtained for publication of all patient photographs. For a full description of the patterns, see the main text

There is a single dorsal cranial field that is centred bilaterally and symmetrically on the midline of the scalp around the vertex (Figure 3a,e).

##### Dorsal non‐segmental pattern—limbs

The four distal limb fields exhibit a glove‐and‐stocking‐type distribution, from above the fingertips up to the shoulder on the upper limb, and from above the toes up to below the knee (Figures 2g–j,m,n and 5c,d). In both upper and lower limbs, the upper cut‐off appears as a transverse line circumferentially across a limb (Figure 2g–j,n) and they appear to develop from dorsal to ventral (Figure 2m). Although these are most commonly seen as unilateral single limb patterns, in the presence of dorsal truncal field involvement, bilateral symmetrical lower limb fields can also be seen in mosaic disorders, with sparing of the mid‐limbs (Gupta et al., 2016; Nanda et al., 2016).

**Figure 4:**
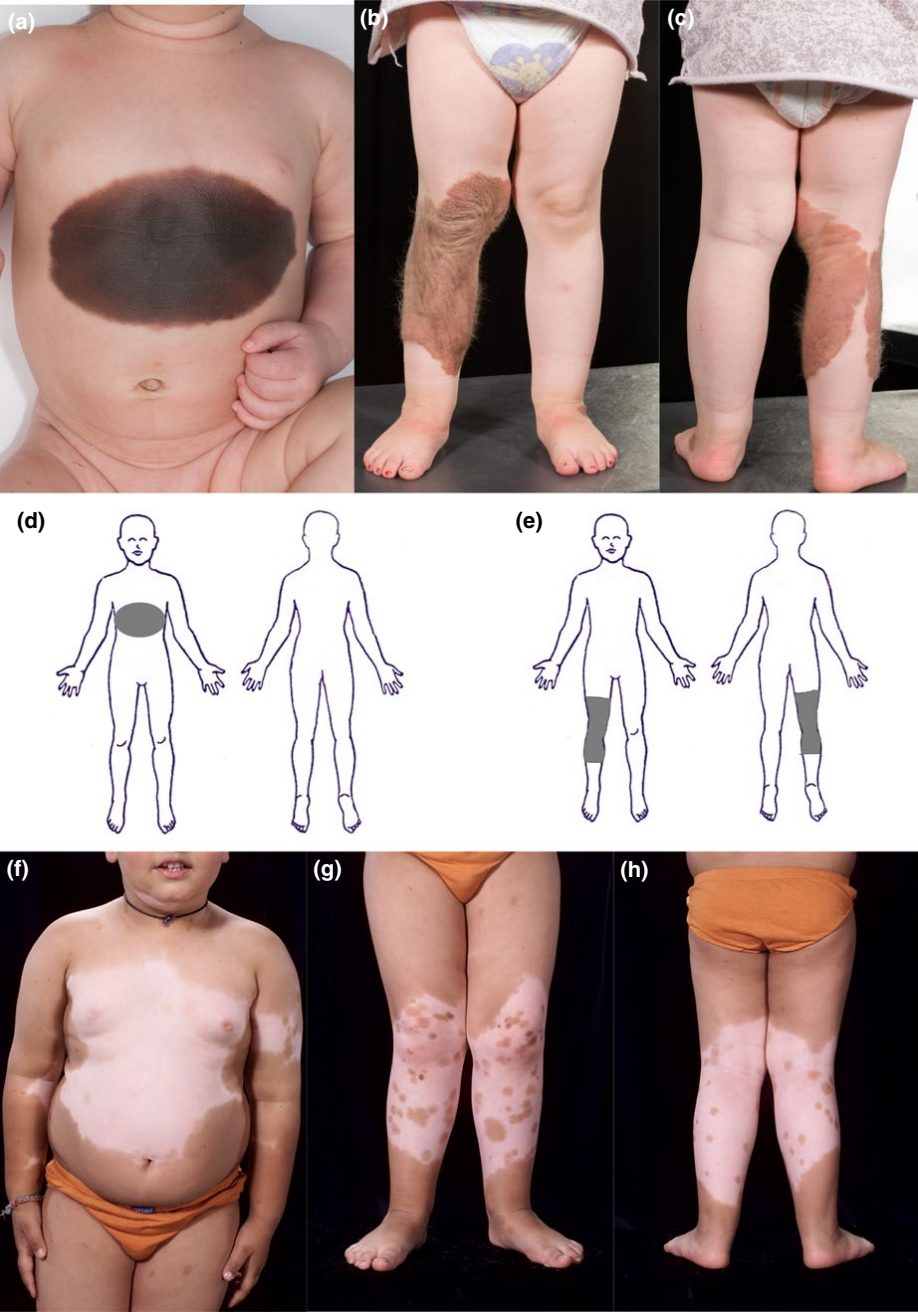
Ventral non‐segmental patterns of pigmentary disorders at the level of the trunk. (a) Occasional occurrence of an anterior/ventral congenital melanocytic naevi (CMN) which spans the anterior/ventral midline but without a directly connected dorsal component, suggesting an anterior/ventral truncal field, and corresponding to the schematic representation of the melanocytic precursor field in (d). (b,c) Mid‐limb circular field CMN which appears to be centred on a dorsal point with ventral extension, corresponding to the schematic representation in (e). (f–h) piebaldism, where the abnormal skin is hypo‐ or depigmented, apparently demonstrating decrease/absence of pigmentation in the distribution of the anterior truncal (f) and mid‐limb fields (g,h), and revealing the bilateral, symmetrical and convex‐outwards patterns of the dorsal cranial, truncal and distal limb fields. Interestingly, this phenotype corresponds to the schematic presented in Figure 5i. Written consent was obtained for publication of all patient photographs. For a full description of the patterns, see the main text

**Figure 5:**
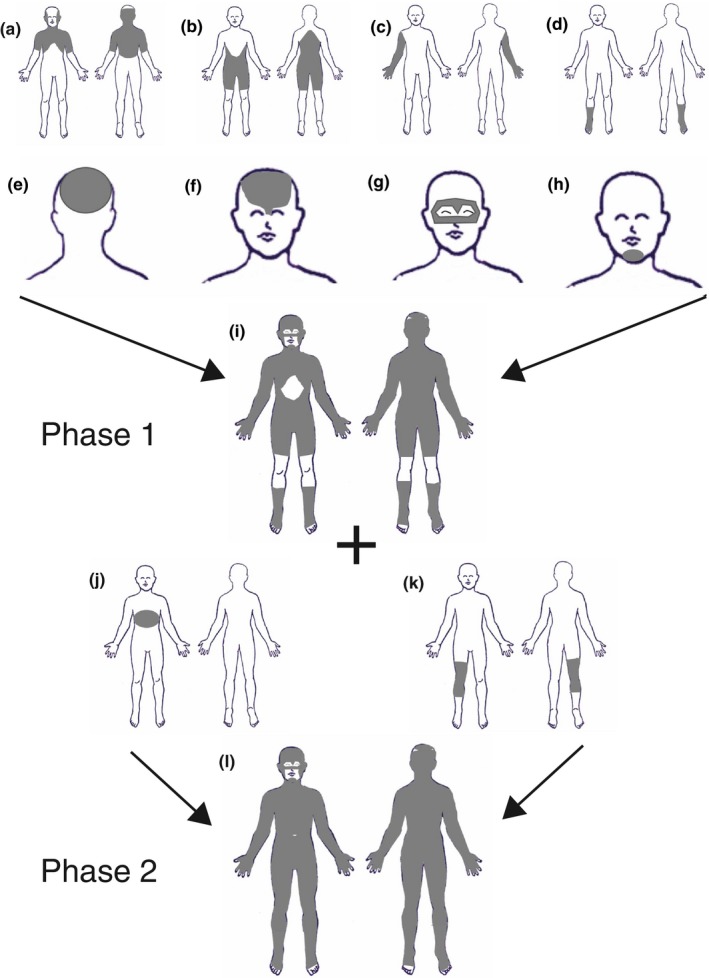
Schematic representation of the major melanocytic precursor fields of the non‐segmental pattern. Individual fields of pigmentation that appear to develop during the first phase, demonstrated in grey. Field for (a) dorsal upper trunk/neck/proximal upper limb (cape distribution), (b) dorsal lower trunk/proximal lower limb (bathing trunk distribution), (c) dorsal distal upper limb, (d) dorsal distal lower limb, (e) midline posterior/dorsal scalp, (f) midline ventral frontonasal, (g) midline mid‐face and (h) midline mandibular regions. (i) Assembly overlap mapping of the fields (a to h) from phase 1. This shows areas of paucity of coverage on the face around the orifices and anterior/ventral abdomen and at the knees anteriorly and posteriorly, as well as at digit tips, which closely resembles the pattern of hypopigmentation seen in piebaldism (Figure 4f,g,h). (j–l) Later fields of pigmentation which develop in phase 2, on the anterior/ventral abdomen (j) and mid lower limbs (l). For a full description of the patterns, see the main text

The two mid‐limb fields are centred on the knees, and extend from the mid‐thigh to mid‐calf (Figures 4b,c,e and 5k). They also have transverse circumferential upper and lower borders and appear to develop from dorsal to ventral. It is possible there are similar mid‐limb fields on the upper limbs, and axillary fields, but there are insufficient data to support this currently. The mid‐limb fields appear later than the distal limb fields and appear to be specified unilaterally.

##### Ventral non‐segmental pattern—trunk and head

Anterior/ventral truncal pigmentary abnormalities are very rarely seen on their own in the absence of posterior/dorsal truncal involvement (Figure 4a,d; Kadhiravan & Sharma, 2009). Where these do arise, they are comparable to the rest of the non‐segmental pattern by their key features (Table 1). This anterior/ventral truncal field is symmetrical and bilateral across the anterior/ventral midline, up to the level of the nipples and above the umbilicus. It may develop after the truncal, cranial and distal limb fields (Figure 5). It is possible that there is a lower abdominal anterior truncal field, but there are insufficient data to support this currently.

The three anterior cranial (facial) fields are bilateral and symmetrical, spanning the anterior/ventral midline, with some degree of overlap between them. The upper midline field extends from the scalp (the exact location is not clear due to the difficulty in visualizing the scalp skin on photographs), onto the central forehead, above and slightly below the medial aspect of the eyes and down onto the bridge of the nose, in a distribution reminiscent of the upper part of the embryonic frontonasal facial placode (Figure 3b,f). The mid‐face field is akin to an eye mask in the centre of the face, extending around both eyes avoiding the edges of the palpebral fissures, and across and around the nose (Figure 3c,g; Natarajan, Arunachalam, Sundar, & Srinivas, 2013). The lower‐face field is currently less clearly defined but appears to be centred on the chin (Figure 3d,f).

##### The “spray can” effect

**Figure 6:**
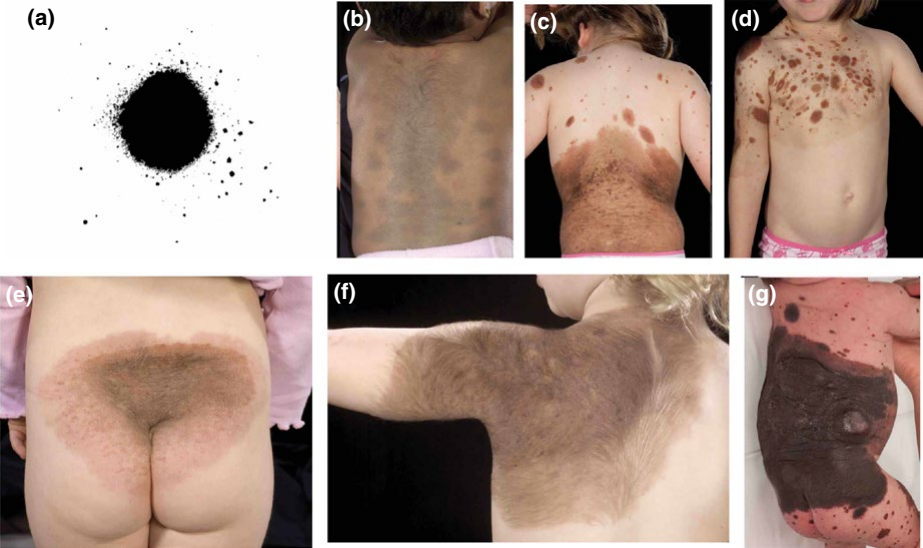
The proposed spray can pattern of melanocytic precursor diffusion in the non‐segmental population. (a) Characteristic scatter of paint from a spray can. (b–d) Examples of melanocytic birthmarks demonstrating a larger, and multiple smaller foci, of pigmentation. (b) Extensive dermal melanocytosis (EDM), (c) congenital melanocytic naevi (CMN) and (d) naevus spilus congenital melanocytic naevus. An example of the spray can pattern visible in piebaldism is shown in Figure 4f–h, revealed by the abnormally hypopigmented areas of skin. (c–d) In some melanocytic birthmarks, smaller foci of pigmentation are also visible within the larger focus. (e–g) Apparently overlaid concentric layers of pigmentation in different CMN, visible as clear steps in intensity of pigmentation, suggestive of successive waves of cell division and diffusion from a central focus. Written consent was obtained for publication of all patient photographs. For a full description of the patterns, see the main text

The “spray can” effect is found in the non‐segmental pattern but not the segmental pattern (Figure 6) The “spray can” epithet is suggested to describe and interpret the frequently observed combination of one larger focus of pigmentation on the skin with smaller foci elsewhere, akin to the pattern of paint which emanates from a spray can (Figure 6a). The sizes of both the larger and smaller foci appear to be independently variable between patients: the large foci covering up to >80% of the body surface area and the smaller foci varying from millimetres to several centimetres in diameter (at birth) depending on the condition. It can sometimes also manifest as multiple smaller foci without one large area. The shape of the foci is generally round or ovoid (Figure 6). Examples of the smaller foci can also be visible within the larger area of pigmentation, depending on the condition (Figure 6c,d,f).

There is sometimes a visible stepwise difference in intensity of pigmentation within the larger focus, from more concentrated in the centre to paler at the edges. This is suggestive of overlaid concentric layers or waves of diffusion, or successive “sprays” from the central focus (Figure 6e–g). The “spray can” effect can also be seen in piebaldism. In this case, the small “sprays”/foci are of normal pigmentation, made visible by the pathogenic lack of background pigmentation in certain areas (Figure 4f–h).

#### 3.1.3 Melanocyte cell‐autonomous patterns with respect to specific structures

##### Digit tips and nail beds

The finger and toe tips and the melanocyte population of the nails are not involved by either the segmental or non‐segmental pattern (Figures 1g–h, 2h,n). Furthermore, in individuals with large pigmented birthmarks and multiple smaller foci elsewhere, the nail bed pigmentation is not usually affected by the smaller foci. This suggests that the nail beds are supplied by their own melanocyte population not addressed here.

##### Palms and soles

The palms and soles can be involved in the non‐segmental pattern, as part of the distal limb field (Figure 2h). In addition, small foci in the spray can pattern can affect palms or soles if the large focus is in any other field. The segmental pattern abnormalities do not involve the palms or soles, despite their ventral/flexor surface coverage of the rest of the limb.

##### The areola of the nipple

**Figure 7:**
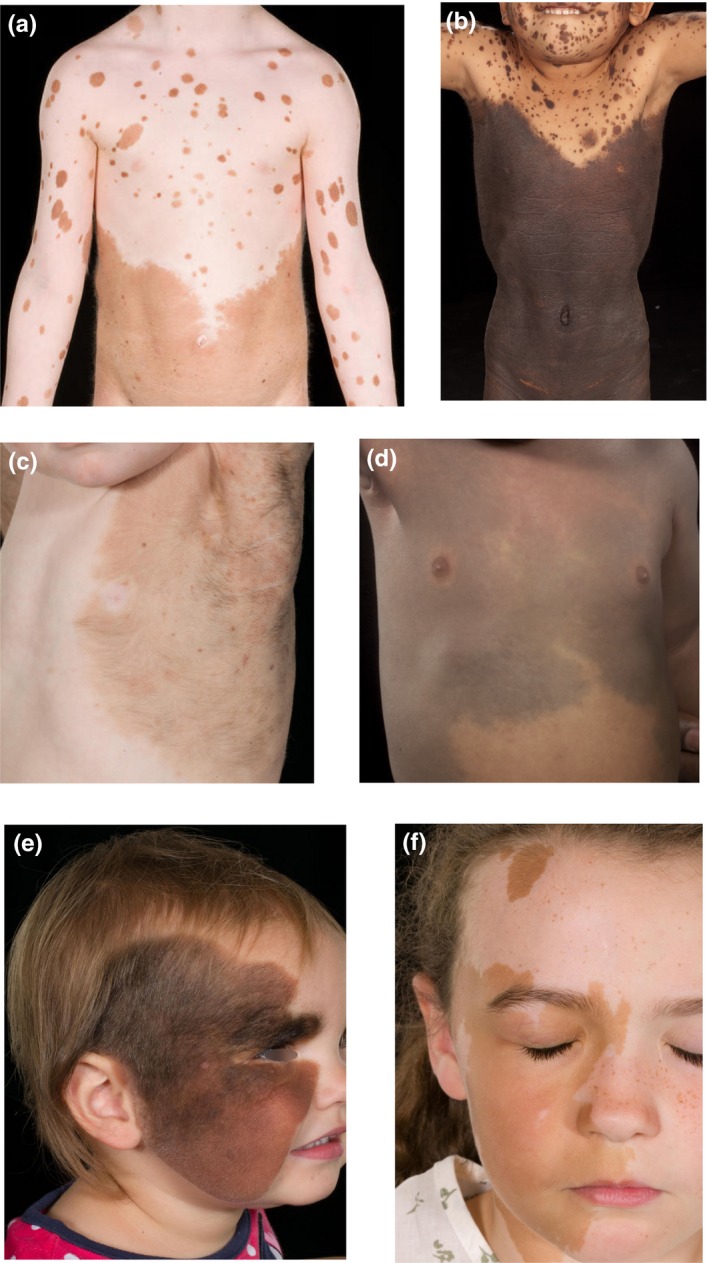
Site‐specific examples of the segmental and non‐segmental patterns in informative structures. (a,b) Variable sparing of the umbilicus in congenital melanocytic naevi (CMN) showing that the umbilicus is pigmented by the non‐segmental population if the level of involvement is high enough on the trunk (B). (a–d) The areolae are usually spared by this population of melanocytic precursors, seen here in CMN (a–c) and phakomatosis pigmentovascularis (PPV) (d); however, the areolae can occasionally be involved but usually this is in association with a deformed nipple structure (b). (e) Non‐segmental pattern CMN (later mutation therefore unilateral) demonstrates sparing of the palpebral fissures and peri‐oral area. (f) By contrast, the segmental pattern melanocytic pigmentation proceeds right up to and including the palpebral fissures and through the vermilion border of the lip. Written consent was obtained for publication of all patient photographs. For a full description of the patterns, see the main text

The non‐segmental pattern frequently spares the areola (Baykal, Solakoglu, Polat Ekinci, & Yazganoglu, 2015), even usually where it is completely surrounded by the pigmentary abnormality in the truncal fields (Figure 7c,d). However, this should not be taken as an absolute rule, as in some cases, the areola is clearly involved in the pigmentary abnormality (Figure 7b; Gangireddy & Coleman, 2013), and in those cases, it may also not properly formed as a structure. The segmental pattern on the other hand does involve the pigmentation of the areola where the surrounding skin is affected (Chebel et al., 2010; Tadini et al., 1998; Tekin, Yucelten, & Happle, 2016).

##### The palpebral fissures and eyelashes, and the vermilion border of the lips

The skin of the palpebral fissures, the eyelashes and the vermilion border of the lips are not usually involved by the non‐segmental pattern melanocytic disorders (Figure 3b, 7e), unlike in the segmental pattern where all can be affected (Figure 7f).

## 4 DISCUSSION

Current knowledge of the dorsolateral pathway only explains one of the two common and recurrent patterns seen in genetically unrelated, congenital, melanocyte cell‐autonomous pigmentary disorders. Here, we propose that the non‐segmental “round‐shape” pattern represents a hitherto‐undescribed population of melanocyte precursors. Caveats surrounding this proposal are that human early embryonic development cannot be studied directly, that we have not taken into account possible effects of interactions between mutant and wild‐type cells in the mosaic clonal disorders, and that there could be an unknown and unsuspected commonality between the genotypically distinct diseases we have studied that is responsible for their identical patterns.

**Figure 8:**
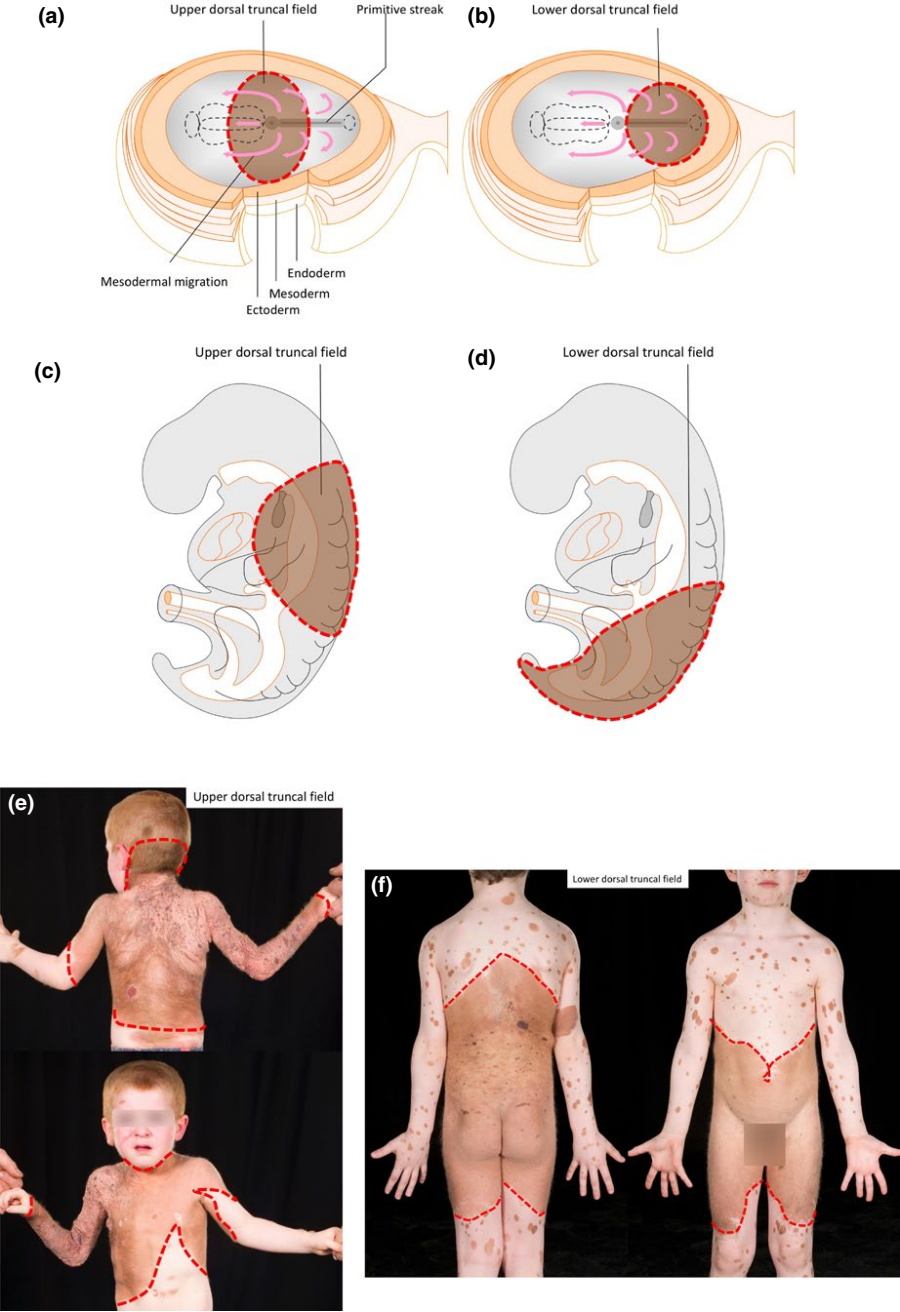
Proposed embryological explanation for the pattern of the dorsal truncal non‐segmental fields. (a,b) Schematic human embryo at the time of gastrulation at the beginning of week 3, showing the approximate origin and initial expansion of the melanocyte precursors for the two dorsal truncal fields in brown, upper (a) and lower (b). (c,d) Schematic human embryo during the process of embryonic folding in week 3, demonstrating the upper (c) and lower (d) truncal fields in brown, which overlap along the dorsal midline, expanding by a proposed passive process of cell division and radial diffusion with growth. (e,f) Clinical examples of extensive congenital melanocytic naevi (CMN) corresponding to the upper (e) and lower (f) dorsal truncal fields. The dotted red lines delineate the edges of the fields, to demonstrate the persistence of the circular migration pattern mapped onto a mature human 3D form. Written consent was obtained for publication of all patient photographs. For a full description of the patterns, see the main text

It is not clear when, and from which germ layer, this novel population arises, and exactly where it ends up. There are however important clues which can lead us to a hypothesis. We can observe that it colonizes the head, trunk and proximal limbs in a bilateral and symmetrical manner and has both dorsal and ventral branches on the head and the trunk. In addition, the pigmentary disorders which display this pattern have predominantly dermal histological pathology, and do not affect key ectodermally derived structures. The non‐segmental population is therefore proposed to fit best with the pattern of migration of mesodermal cells arising in the area of the primitive streak around the time of gastrulation and that the population enlarges outwards randomly, by a process of cell division and centrifugal diffusion with the growth of the embryo, bilaterally and symmetrically along the full length of the embryo (Figure 8). We also propose that it ultimately resides within the dermis. It is however also conceivably compatible with a very early ectodermal population, arising before gastrulation and the division of the embryo into right and left, providing this is distinct from the neural crest dorsolateral population. This theory could be tested by lineage tracing in, for example, mouse embryos, by labelling mesodermal cells with a fluorescent or similar marker coupled to expression of a melanoblast‐specific gene, and looking within the dermis post‐natally for evidence of labelled cells. It could also be tested by multiple single‐cell DNA sequencing, leading to phylogenetic tree construction of cellular development, from human subjects with a known causative mutation, by establishing the timing of the mutation embryologically and the lineage of that cell. This would potentially use somatic mutational profiling previously established for embryological lineage tracing from the adult human (Ju et al., 2017).

In some mosaic clonal disorders, there is clearly a single dorsal truncal field, suggesting that there is originally a single truncal melanocyte precursor cell which is first specified in the dorsal midline (at approximately day 15 human, E6.5 mouse) and whose progeny populate the whole of the dorsal trunk with variable extension ventrally, and the proximal limbs (i.e., the upper arms and the thighs, before these are visibly formed as separate structures). On the basis of the recurrent pattern of upper and lower dorsal truncal fields seen separately in mosaic clonal disorders, this cell then appears to divide into two, to be centred over the cranial and caudal ends of the developing spine. If we were able to visualize first one and then the other cell's offspring and their pigmentation, what we would expect to see on the flat disc would be a circular/ovoid population at the cranial or caudal end (Figure 8a,b). Imagining this circular lesion translated onto the newborn human, after lateral embryonic folding and the development of head and limbs (Figure 8c,d), it can be seen that this correlates with the post‐natal phenotype in different mosaic clonal disorders (Figures 2a–f, 5a–b and 8e–f). At a later embryonic stage (estimated week 4 human, E10‐11 mouse), non‐segmental melanocyte precursors appear within the developing head (Figure 3) and in the limb buds. Whether the founder cells for the developing head and limb buds are specified before the development of the face and limb buds is unknown; however, data from rare cases of CMN and EDM where one or both distal limb fields can be involved as well as the caudal truncal field (Gupta et al., 2016; Nanda et al., 2016), or facial fields as well as cranial and caudal truncal fields (Raina & Chaudhuri, 2010; Ramesh et al., 2017), support this hypothesis. The alternative would be that the non‐segmental melanocyte precursor for each of these fields arises independently in that new structure.

Fascinatingly, the non‐segmental pattern fields are also observable in piebaldism; however, in this case, the abnormal skin is hypo‐ or depigmented, and it is the normal skin that delineates the upper and lower dorsal truncal fields, and distal limb fields (Figure 4f–h). This germline condition has been shown in mice to cause a reduction in numbers of dorsolateral melanoblasts with an increase in their diffusion rate (Mort et al., 2016). It is also potentially possible that the *KIT* mutation affects certain melanocyte populations more than others, for example, the ventral truncal and mid‐limb fields of the non‐segmental population (Figure 4a–d) with variations in phenotypic severity due to the exact mutation and other germline modifiers.

The terminology here is difficult to define from these observations alone. We are proposing that ultimately, a single cell gives rise to a whole population of melanocyte precursors, however hesitate to term it a melanoblast. Diseases causing pigmentary patterns corresponding to the single‐cell stage (Figure 2k,l) can also give rise to abnormalities of the melanocytes of the eye, and blood vessels of the skin, eye and brain, but these are not universal. At the stage when we can map the individual fields as detailed in Figures 2a–l, 3 and 5, there can be congenital abnormalities of the brain parenchyma and/or leptomeninges; however, these are only seen in a minority of cases.

The segmental patterns of pigmentation appear to correlate with what is currently known of the dorsolateral melanoblast population. Alternating mosaic and proven clonal patterns from a single post‐zygotic mutation, such as the checkerboard pattern in PPK or MAS, directly infer some cranio‐caudal axial mixing of the dorsal melanocyte precursors before migration, already documented in this population in mice (Wilkie, Jordan, & Jackson, 2002). Occasionally, these segmental patterns appear truly bilateral at one cranio‐caudal level (Figure 1d). Whether this is truly a representation of a single precursor cell leading to bilateral migration from the neural crest, or simply a version of the checkerboard pattern which by chance occurred at the same cranio‐caudal segment is not clear, but the same occasional occurrences were described in early murine chimaeric experiments (Mintz, 1965).

As regards the number of single melanocytic precursors in the segmental population, the estimate in the current study is approximately 12–16 (Table 1, Figure 1g,h), on the basis of the mosaic clonal patterns in humans, at a time where the patterns can occur without involvement of other organ systems. This and all other features of the segmental population described here would be compatible with the conclusions of studies of mouse chimaeric experiments (Mintz, 1967) and later mosaic modelling of melanocyte migration (Huszar et al., 1991). These two studies also concluded that there are very few “founder melanoblasts,” although this has been disputed by subsequent studies (Mort et al., 2016; Wilkie et al., 2002). Interestingly, a recent estimate of the number of founder cells for the entire blood lineage is of a similar order, at 10 (Rahbari et al., 2016). We suggest that these apparently conflicting findings in numbers of melanoblasts could in fact be compatible with each other and may be due to differences in definition of the word “melanoblast.” There may be a small number of earlier dorsolateral/segmental population founder precursor cells giving rise to a larger number of what are defined molecularly as melanoblasts in the mouse at E11.5. It may also be, however, that the existence of the proposed non‐segmental population of melanocyte precursors confounds mathematical modelling of final melanocyte numbers in the skin, particularly if this population only becomes apparent by classical melanoblast labels at a certain embryonic stage.

The absence of known melanocytic disorders in a fine and whorled Blaschko linear suggests that this pattern of pigmentation is non‐cell‐autonomous, the result of mosaicism or chimaerism affecting keratinocytes (either at DNA or RNA level), leading to a pigmentary phenotype. This would be in line with the basis for the brindle pattern in dogs, proposed to be dictated at the level of keratinocytes not melanocytes (Kerns et al., 2007), and with recent evidence from a mosaic KITLG mutation in one patient (Sorlin et al., 2017). This will be testable by single‐cell analysis in patients with this phenotype and in whom the causative mosaic or chimaeric mutation has been established.

The question then arises as to which mature melanocytes the segmental and non‐segmental populations relate to at birth. Patterns related to specific structures can potentially be informative in addressing this, and in helping deduce the timing of development. The segmental patterns of pigmentation are demonstrably associated with the epidermis, given the noticeable disregard for the boundaries at the areola, the lips and the eyelids, with the pigmentary abnormality proceeding right through these structures (Figures 1a and 7f). This suggests that the epidermis had already developed or was developing at the same time as these melanocyte precursors. An elegant supporting observation is that segmental pattern pigmentary birthmarks take the place of freckles (normal ephelides) in individuals who have them (Figures 1a and 7f). This is in sharp contrast to what is usually seen with the non‐segmental pattern where the boundaries of these epidermal structures act as an interruption to pigmentation, strongly suggesting that the epidermal structures developed at a later stage or at least independently of the non‐segmental melanocyte population. A correlate of this is that non‐segmental disorders take the place of mongolian blue spots where these abut each other.

Perhaps the most important potential sequela of identifying these two melanocyte cell‐autonomous patterns is an improved understanding of acquired pigmentary diseases. There are, for example, striking parallels with the patterning of segmental and non‐segmental vitiligo (Ezzedine, Eleftheriadou, Whitton, & Van Geel, 2015; Picardo et al., 2015). In non‐segmental vitiligo, depigmentation tends to start in what correlates to the areas of paucity of the non‐segmental fields shown in Figure 5i. Furthermore, the melanocyte populations of the eyelashes and eyebrows are typically unaffected in early disease, as are the areolae of the nipples and the lips (Faria, Tarle, Dellatorre, Mira, & Castro, 2014). In contrast, the affected areas in segmental vitiligo closely parallel, and are restricted to, the segmental pattern of melanocytic disorders described here, and in the appropriate area will involve the epidermal structures of the eyelashes, eyebrows, areolae and lips from early in the disease (Faria et al., 2014). These subtypes of vitiligo can therefore be proposed as diseases of different melanocyte populations, independent of the disease mechanism itself.

In conclusion, we propose that a novel population of melanocyte precursors arises around the time of gastrulation within the mesoderm and migrates centrifugally by proliferation and diffusion from dorsal and ventral midline foci to take up residence in the dermis. It is not known whether this population contributes to normal pigmentation, or whether melanocytic differentiation is only seen in congenital genetic disease conditions. However, given the patterns seen in vitiligo, we suggest that this new population does contribute to normal pigmentation of the skin in humans, and most likely in other vertebrates.
