# Supplementary material for: The patterns of birthmarks suggest a novel population of melanocyte precursors arising around the time of gastrulation
Source: Pigment Cell Melanoma Res. 2017 Oct 13;31(1):95–109. doi: 10.1111/pcmr.12645 (PMC5765478; doi:10.1111/pcmr.12645)
Supplement: Supplementary file 1 [file PCMR-31-95-s001.docx]

## **Supplementary material**

## *Pigmentary disorders referred to frequently in this work*

Piebaldism (Figures 1,2) is a rare disorder caused by a heterozygous germline mutation in the genes *KIT* (Spritz et al., 1992) or *SNAI2* (Sanchez-Martin et al., 2003), inherited in an autosomal dominant manner. This condition is characterized by variable degrees of hypo- or depigmentation, bilaterally and more or less symmetrically in a primarily anterior midline distribution on the scalp, face and trunk, and on the limbs usually centred around the knees and elbows. Classically there are what are termed ‘café-au-lait macules’ within the areas of depigmentation, and sometimes hyperpigmented macules. The cutaneous findings in this condition mirror those of the murine dominant White Spotting phenotype, caused by defects in the gene homologous to *KIT*, which has recently been demonstrated to be a cell-autonomous melanocytic defect (Aoki et al., 2015).

Congenital Melanocytic Naevus (CMN) syndrome (Figures 1-5) is a rare disorder usually caused by a heterozygous post-zygotic mutation in the gene *NRAS* (Kinsler et al., 2013), and therefore sporadic rather than inherited. This condition is usually characterized by one much larger melanocytic naevus, and a variable number of other smaller naevi. It is known that a single mutation gives rise to both the large and small naevi (Kinsler et al., 2013), and therefore that the distribution is the result of the offspring of one melanocyte precursor. The pattern of pigmentation depends on the principles governing mosaic phenotypes above.

Phakomatosis Pigmentovascularis (PPV) is a rare disorder usually caused by a post-zygotic mutation in the homologous genes *GNA11* or *GNAQ* (Thomas et al., 2016), and is therefore also sporadic. This condition is characterized by the combination of pigmented and vascular birthmarks, and in those where the gene has been identified as above are a combination of extensive dermal melanocytosis (Mongolian blue spots but which are generally extensive and persistent) and vascular birthmarks known as capillary malformations. It is known that a single mutation gives rise to both the pigmentary and vascular birthmarks (Thomas et al., 2016), inferring a common melanocytic and vascular precursor. Again the pattern varies dependent on the principles governing mosaic phenotypes. PPV is in the middle of a spectrum of conditions caused by the same mutations, with Sturge-Weber syndrome at one extreme producing only the vascular phenotype, PPV in the middle producing vascular and pigmentary, and Extensive Dermal Melanocytosis (EDM, Figures 2,5,6) at the other extreme producing only the pigmentary phenotype (Thomas et al., 2016). Naevus of Ota and Naevus of Ito are the same types of birthmarks and can occur as part of the pigmentary phenotype of PPV or EDM.

Phakomatosis Pigmentokeratotica (PPK) is a rare disorder which in the few cases described is caused by a post-zygotic mutation in the gene *HRAS* (Groesser et al., 2013), therefore sporadic in occurrence. This condition is characterized by the combination of epidermal naevi (either keratinocytic or sebaceous or a mixture) and pigmented birthmarks (classically naevus spilus papulosus – a café-au-lait macular background with smaller melanocytic proliferations within it). Again both birthmarks have been shown to be caused by the same mutation, pointing to the existence of a common precursor for melanocytic and epidermal cells. The mutation has been isolated from the melanocytes of the naevus spilus (Groesser et al., 2013).

McCune-Albright syndrome is a rare disorder caused by a post-zygotic mutation in the gene *GNAS* (Weinstein et al., 1991), and is therefore sporadic in occurrence. It is characterized from the cutaneous viewpoint by large areas of café-au-lait macular pigmentation with a sharp midline cutoff, and classically by waviness of the edges likened to the Coast of Maine, although alternating unilateral or bilateral broad Blaschko-linear pigmentation has also been described. The mutation has been isolated from melanocytes in the café-au-lait macules (Kim et al., 1999).

Neurofibromatosis type 1 is a rare disorder caused by germline mutations in the gene *NF1* (Wallace et al., 1990), inherited as an autosomal dominant condition in approximately 50% of cases, and arising *de novo* in the other 50%. It can also occur as a post-zygotic mosaic phenomenon with only part of the body affected. The characteristic pigmentary skin findings are multiple café-au-lait macules, which are sometimes present at birth and can be large if so, or more usually appear over years after birth and are classically small and ovoid, with smoother edges.

The last condition is Vitiligo, which in contrast to the other diseases here is relatively common and acquired, and for which no single gene defect has been identified. It is characterized by melanocyte loss, and a large variety of aetiological mechanisms have been proposed. The pattern of this disease in its classical form is a bilateral and largely symmetrical depigmentation with a particular predilection for a certain distribution in its early stages, notably the face, particularly peri-orificially, the anterior chest and abdomen, the knees and elbows, hands and feet. In the segmental form of vitiligo only a part of the skin is affected, and a sharp midline cutoff is respected. This form has some distinct features from the classical form in terms of epidemiology and natural history.

Aoki, H., Tomita, H., Hara, A., and Kunisada, T. (2015). Conditional Deletion of Kit in Melanocytes: White Spotting Phenotype Is Cell Autonomous. The Journal of investigative dermatology.

Groesser, L., Herschberger, E., Sagrera, A., Shwayder, T., Flux, K., Ehmann, L., Wollenberg, A., Torrelo, A., Bagazgoitia, L., Diaz-Ley, B., et al. (2013). Phacomatosis pigmentokeratotica is caused by a postzygotic HRAS mutation in a multipotent progenitor cell. The Journal of investigative dermatology *133***,** 1998-2003.

Kim, I. S., Kim, E. R., Nam, H. J., Chin, M. O., Moon, Y. H., Oh, M. R., Yeo, U. C., Song, S. M., Kim, J. S., Uhm, M. R., et al. (1999). Activating mutation of GS alpha in McCune-Albright syndrome causes skin pigmentation by tyrosinase gene activation on affected melanocytes. Hormone research *52***,** 235-40.

Kinsler, V. A., Thomas, A. C., Ishida, M., Bulstrode, N. W., Loughlin, S., Hing, S., Chalker, J., Mckenzie, K., Abu-Amero, S., Slater, O., et al. (2013). Multiple congenital melanocytic nevi and neurocutaneous melanosis are caused by postzygotic mutations in codon 61 of NRAS. The Journal of investigative dermatology *133***,** 2229-36.

Sanchez-Martin, M., Perez-Losada, J., Rodriguez-Garcia, A., Gonzalez-Sanchez, B., Korf, B. R., Kuster, W., Moss, C., Spritz, R. A., and Sanchez-Garcia, I. (2003). Deletion of the SLUG (SNAI2) gene results in human piebaldism. American journal of medical genetics. Part A *122A***,** 125-32.

Spritz, R. A., Giebel, L. B., and Holmes, S. A. (1992). Dominant negative and loss of function mutations of the c-kit (mast/stem cell growth factor receptor) proto-oncogene in human piebaldism. American journal of human genetics *50***,** 261-9.

Thomas, A. C., Zeng, Z., Riviere, J. B., O'shaughnessy, R., Al-Olabi, L., St-Onge, J., Atherton, D. J., Aubert, H., Bagazgoitia, L., Barbarot, S., et al. (2016). Mosaic Activating Mutations in GNA11 and GNAQ Are Associated with Phakomatosis Pigmentovascularis and Extensive Dermal Melanocytosis. The Journal of investigative dermatology.

Wallace, M. R., Marchuk, D. A., Andersen, L. B., Letcher, R., Odeh, H. M., Saulino, A. M., Fountain, J. W., Brereton, A., Nicholson, J., Mitchell, A. L., et al. (1990). Type 1 neurofibromatosis gene: identification of a large transcript disrupted in three NF1 patients. Science *249***,** 181-6.

Weinstein, L. S., Shenker, A., Gejman, P. V., Merino, M. J., Friedman, E., and Spiegel, A. M. (1991). Activating mutations of the stimulatory G protein in the McCune-Albright syndrome. The New England journal of medicine *325***,** 1688-95.
